# Practical considerations in the calibration of CT scanners for proton therapy

**DOI:** 10.1120/jacmp.v15i3.4721

**Published:** 2014-05-08

**Authors:** Christopher G. Ainsley, Caitlyn M. Yeager

**Affiliations:** ^1^ Department of Radiation Oncology University of Pennsylvania Philadelphia PA USA

**Keywords:** proton therapy, CT calibration, range uncertainty

## Abstract

Treatment planning systems for proton therapy require a CT calibration curve relating Hounsfield units to proton stopping powers. An understanding of the accuracy of this curve, together with its limitations, is of utmost importance because the calibration underpins the calculated dose distribution of every patient preparing to undergo proton therapy, independent of delivery technique. The most common approach to the calibration is the stoichiometric method, which is well‐defined and, in principle, straightforward to perform. Nevertheless, care must be taken when implementing it in the clinic in order to avoid introducing proton range uncertainties into treatment plans that are larger than the 3.5% that target margins are typically designed to account for. This work presents a variety of aspects related to the user‐specific implementation of the stoichiometric calibration, from both a measurement setup and a data‐handling point of view, and evaluates the potential impact of each for treatment planning purposes. We demonstrate that two alternative commercial vendors' tissue phantoms yield consistent results, that variable CT slice thickness is unimportant, and that, for a given cross‐sectional size, all phantom data can, with today's state‐of‐the‐art beam hardening‐related artifact reduction software, be acquired quickly and easily with a single scan, such that the resulting curve describes the calibration well at different positions across the imaging plane. We also show that one should be cautious of using metals in the calibration procedure and of using a single curve for anatomical sites differing widely in size. Further, we suggest that the quality of the parametric fit to the measurement data can be improved by performing a constrained, weighted linear regression. These observations, based on the 40 separate curves that were calculated, should help the medical physicist at any new proton therapy facility in deciding which considerations are worth particular attention.

PACS numbers: 87.53.Bn, 87.55.‐x, 87.57.Q‐, 87.59.bd

## I. INTRODUCTION

Protons of therapeutic energy passing through tissue slow down continuously, primarily via inelastic collisions with atomic electrons, until eventually reaching a halt. The rate of energy deposition increases with depth and is most dramatic in the “Bragg peak” near the end of the protons' range.^(1)^ The relationship between range and incident beam energy is well‐defined, which means that this range can be matched to the targeted depth by appropriate selection of beam energy. Such characteristics provide proton therapy with a distinct physical advantage over conventional X‐ray therapy; the gradual buildup of dose prior to the dramatic increase within the target, coupled with a lack of exit dose, leads to overall less ionization energy being deposited in healthy tissues for the same prescribed dose to the target.

The calculation of proton therapy dose distributions typically relies on knowledge of the three‐dimensional map of relative (to water) proton stopping powers throughout the parts of the patient anatomy through which the beam passes. While this could be derived directly from computed tomography (CT) images acquired in a proton beam, such proton CT scanners have yet to become commercially viable, although technical developments are ongoing.^2^, ^3^, ^4^, ^5^, ^6^, ^7^, ^8^, ^9^, ^10^, ^11^ Consequently, this map must currently be constructed from X‐ray CT images, and the information contained therein transformed in some indirect manner to relative proton stopping powers. The stoichiometric method is the technique used most widely for performing this transformation.^12^, ^13^ While this method has proven to be reliable, it is ultimately limited by the fact that the physics of X‐ray and of proton interactions in matter are fundamentally different, and the mapping is therefore not strictly one‐to‐one. Many of the uncertainties associated with its application have been studied, and there is consensus in the community that in most practical situations this translates into a relative uncertainty in the proton range of ~ 3.5% at the 95% confidence level.^14^, ^15^, ^16^ Margins along the beam direction are added routinely to the clinical target volume in treatment planning to allow for this.

Proton therapy centers are undoubtedly on the increase. For example, in the United States alone, where there are currently 12 centers in operation,^(17)^ a further 13 are either now in the planning stage or are under construction.^(18)^ While there is diversity in technologies, delivery techniques, and treatment planning systems, the CT scanner calibration, which maps Hounsfield units (HUs) to proton stopping powers, is an aspect that is common to all. The last of these is, therefore, of widespread interest, especially to those performing the calibration for the first time. During the undertaking of the stoichiometric calibration at our facility, we found ourselves confronting various practical considerations related to the acquisition and handling of the measurement data. We have not seen most of these addressed previously, and this led us to question whether or not the choice to use 3.5% as the assigned range uncertainty for treatment planning purposes is actually appropriate for our institution's own particular calibration curve. We hope that by answering these in the present work, we will help to dispel similar concerns that physicists exercising the calibration at other centers may have in future.

The stoichiometric calibration is typically carried out with the aid of a tissue characterization phantom, comprising tissue‐surrogate “plugs” of known elemental composition and electron density. Compositional details can be obtained from the commercial vendor, but often these details are nominal and there can be batch‐to‐batch variability. While the ultimate test of the calibration curve is to make direct measurements of points along it with real mammalian tissue samples,^(13)^ such measurements are not easy to perform with sufficient precision. Implicit reliance is therefore placed on the accuracy of the vendor's data tables. To gain assurance of the accuracy of these tables, separate calibrations were performed with tissue surrogates from two independent sources. One set of tissue surrogates was tested further by replacing the nominal electron densities of the plugs provided by the vendor with, instead, values calculated from measurements of their densities, combined with the given elemental composition data. With this set, the impact of CT slice thickness, phantom size, position of the plug within the phantom, and whether the plugs were scanned one‐at‐a‐time at the center of a water phantom or scanned (more conveniently) simultaneously in a solid water phantom holder, were all assessed, in addition. With both sets, the effect of performing a weighted linear regression based on the uncertainties in the measured HUs of the plugs versus an unweighted linear regression to extract the parameters that characterize the CT scanner was explored. Since various metallic plugs are offered by the tissue phantom vendors as optional extras, but their suitability for use in the stoichiometric calibration is questionable, any bias in the result introduced by the inclusion of an aluminum plug was studied. Furthermore, if the outcome was affected by placing a constraint on the standard stoichiometric fit such that only two instead of three parameters^12^, ^13^ were independent was investigated. The sensitivity of the calibration curve to phantom size and plug position during measurements as a result of differential beam hardening has been discussed previously;^13^, ^16^, ^19^, ^20^ to the best of our knowledge, the other considerations identified above have not.

The focus of this work was not to undertake a comprehensive assessment of all of the uncertainties attributable to the stoichiometric calibration, but rather to isolate just those components, alluded to above, that relate to the acquisition and handling of the measurements made with a particular user's CT scanner. Armed with an appreciation of these, future users can perform their calibrations confident in the knowledge of the details, of those that are within their control, that deserve particular attention. In so doing, the user‐dependent uncertainties introduced can be maintained sufficiently small compared to those that are user‐independent such that the combined 3.5% range uncertainty assigned via margins in treatment planning is, to the greatest extent possible, not being underestimated through an institution's specific implementation of the procedure.

## II. MATERIALS AND METHODS

**Figure 1:**
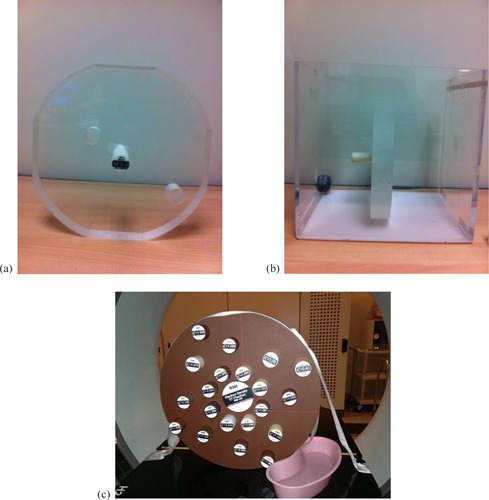
Measurement setup showing (a) the home‐made acrylic holder with holes drilled at the center and at two off‐axis radii (6 cm (”inner hole”) and 12 cm (”outer hole”)), (b) a single plug placed in this holder, positioned in the water tank, and (c) the Gammex tissue characterization phantom with multiple plugs being scanned simultaneously.

The HUs of 14 tissue‐surrogate plugs from one tissue characterization phantom (Gammex 465; Gammex Inc., Middleton, WI) (Table 1), nine tissue‐surrogate plugs from a second phantom (Model 062; CIRS, Norfolk, VA) (Table 1), an aluminum plug and water were measured for a variety of different setups, detailed further below, using a Siemens Somatom Sensation CT scanner (Siemens AG, Erlangen, Germany) with the following, fixed settings: current×time=280mAs (effective), peak X‐ray energy=120kV,SFOV=50cm,matrix size=512×512pixels, pixel size=0.98mm,CTDI=25mGy(32cm diameter phantom),reconstruction filter=B31s. Scans of length 10 cm were performed along the long axis of each cylindrically shaped plug (or similarly sized volume of water), centered on the center of each. In all but one instance, the plugs were positioned in one of three holes drilled at different radii from the central axis in a home‐made acrylic holder and scanned one at a time in a $30\times 40\times 30\;{\text{cm}}^{3}$ water‐filled tank (Figs. 1(a) and (b)). In these cases, each plug was set to protrude far enough from the holder such that the HU recorded at the center of the plug was not affected by any edge effects arising from the boundary between the holder and the water (as assessed by comparing with the HUs recorded in slices 9 mm either side of the central plane). Each plug was also taped securely in place to prevent any movement with respect to the surrounding water as the table translated during the scan. In the remaining instance, the HUs of all plugs were measured simultaneously in a single scan, using the vendor's phantom holder with the plugs distributed across the field of view (Fig. 1(c)). Of all the series of scans above, all but one was reconstructed with 3 mm slice thickness; the other being reconstructed with 1.5 mm slice thickness. We refer to our “standard configuration” as that in which the narrowest dimension of the water tank (30 cm) is aligned with the scan plane, the plugs are placed in turn in the central hole of the holder, and the slice thickness is 3 mm.

**Table 1:** Tissue surrogates employed in this study (using vendors' nomenclature) and their stated relative electron densities

*Gammex*		*CIRS*	
*Description*	$\rho_{e}^{rel}$	*Description*	$\rho_{e}^{rel}$
lung (LN‐300)	0.280	lung (inhale)	0.190
lung (LN‐450)	0.400	lung (exhale)	0.489
adipose (AP6)	0.895	adipose	0.949
polyethylene	0.945	breast (50% gland / 50% adipose)	0.976
solid water	1.000	plastic water	1.002
brain (SR2)	1.039	muscle	1.043
liver (LV1)	1.050	liver	1.052
CB4 resin	1.116	trabecular bone ($200\;\text{mg}\;{\text{cm}}^{-3}$)	1.117
bone (CB2‐10% CaCO_3_)	1.142	dense bone ($800\;\text{mg}\;{\text{cm}}^{-3}$)	1.456
acrylic	1.147		
inner bone (IB)	1.081		
bone (CB2 ‐ 30% CaCO_3_)	1.285		
bone (CB2 ‐ 50% CaCO_3_)	1.473		
cortical bone (SB3)^3^	1.707		

The data were processed in a variety of ways, also detailed further below. In each scenario, the measured HUs of the series of plugs interrogated (with or without their measurement uncertainties, as appropriate) were used to extract the coefficients *A, B*, and *C* — which parameterize the contributions to the linear attenuation coefficient of the CT scanner from the photoelectric effect, coherent scattering, and Compton interactions, respectively — from a linear regression fit to^12^, ^13^, ^21^, ^22^

(1) $$HU_{i}+1000=1000\times \mu_{i}^{rel}=A(\rho_{e_{i}}^{rel}{\tilde{Z}}_{i}^{3.62})+B(\rho_{e_{i}}^{rel}{\tilde{Z}}_{i}^{1.86})+C(\rho_{e_{i}}^{rel})$$

across all plugs, *i*. Here, $\mu_{i}^{rel}$ and $\rho_{e_{i}}^{rel}$ are the linear attenuation coefficient and volumetric electron density relative to water, respectively, of plug $i,{\tilde{Z}}_{i}$ and ${\hat{Z}}_{i}$ are effective atomic numbers defined by

(2) $${\tilde{Z}}_{i}={\left[\sum_{j}\lambda_{j}Z_{j}^{3.62}\right]}^{1/3.62}$$

and

(3) $${\hat{Z}}_{i}={\left[\sum_{j}\lambda_{j}Z_{j}^{1.86}\right]}^{1/1.86}$$

where $\lambda_{j}=\;\frac{w_{j}Z_{j}}{A_{j}}/\sum_{j}\frac{w_{j}Z_{j}}{A_{j}}$ and *w_j_, Z_j_* and $A_{j}$ are, respectively, the mass fraction, atomic number, and mass number of element *j* in the mixture from which plug *i* is composed. These parameters were then used to calculate theoretical Hounsfield units, ${\mathrm{HU}}_{\text{calc}}$, in each scenario for 63 human tissues of known composition taken from the literature.^23^, ^24^ The values obtained were plotted against theoretical relative proton stopping powers, $\rho_{s}^{rel}$, calculated according to the Bethe‐Bloch equation:^(25)^

(4) $$\rho_{s}^{rel}=\rho_{e}^{rel}\times \frac{\text{ln}[2m_{e}c^{2}\beta^{2}/(I_{t}(1-\beta^{2}))-\beta^{2}]}{\text{ln}[2m_{e}c^{2}\beta^{2}/(I_{w}(1-\beta^{2}))-\beta^{2}]}$$

where $m_{e}c^{2}$ is the rest mass energy of the electron, β is the proton speed relative to the speed of light, *c*, and $I_{t}$ and $I_{w}$ are the mean ionization potentials^(26)^ of the tissue in question and water, respectively. The effect of variations in the value of β as a proton beam passes through matter and uncertainties in the *I* values introduce uncertainties in the calibration procedure has been assessed before.^16^, ^27^ However, as these uncertainties are independent of the user while the focus of this study is those which are user‐dependent, they are not considered further here. A fixed proton energy of 115 MeV was used to set the value for β in all cases, close to the energy recommended to minimize the consequence of its variation in practice.^(16)^ Of the 63 tissues, separate linear fits were made through 42 identified as being “organ‐like” in their composition, seven identified as being “fat‐like”, and the remaining 14 identified as being “bone‐like”, with the organ‐like fit constrained to pass through $\rho_{s}^{rel}=0$ at $HU_{\text{calc}}=-1000$. These fits were connected piecewise in regions generally populated sparsely with tissues^(13)^ in order to create the full calibration curve in a particular scenario. Thus, in each case, the organ‐like fit was tracked from ‐1000 to ‐200 HU, the fat‐like fit was tracked from ‐120 to ‐20 HU, the organ‐like fit was tracked again from +35 to +100 HU, and the bone‐like fit was tracked above 140 HU. In all, 40 curves were calculated under different scenarios using the different measurement setups and data‐handling techniques described below. These were intercompared by evaluating the relative difference in $\rho_{s}^{rel}$ (and, hence, water‐equivalent range) between pairs of curves as a function of ${\mathrm{HU}}_{\text{calc}}$.

### A. Extraction of HU data

**Figure 2:**
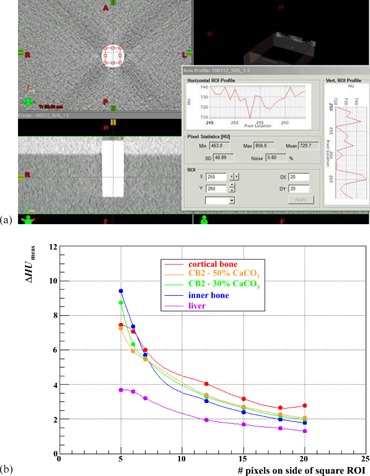
(a) Determination of the standard error on the mean HU, $\Delta HU_{\text{meas}}$. of the Gammex bone (CB2 $‐30\;\%{\text{CaCO}}_{3}$) plug for an ROI of 20 pixels×20 pixels, and (b) variation of this error with the size of the ROI for this and for four of the other plugs, listed in order of descending HU.

Regions of interest (ROIs) of different square size, centered on the axis of each plug scanned, were identified in the CT data (Fig. 2(a)) and, in each instance, the standard error on the mean HU of the plug, $\Delta HU_{\text{meas}}$, in the slice used for analysis was calculated (e.g., for the bone plug shown in Fig. 2(a) with an ROI of 20 (”DX”) ×20 (”DY”) pixels and a standard deviation on the measured HU of 40.89 (”SD”), $\Delta HU_{\text{meas}}=\text{SD}/\sqrt{(}\text{DX}\times \text{DY})=2.0$, as plotted in Fig. 2(b)). The ROI for which this quantity was minimized was used subsequently.

### B. Electron density

The HU of each of the 14 Gammex plugs was measured in the standard configuration (narrowest tank dimension aligned with the scan plane; plugs placed in turn in the central hole of the holder; 3 mm slice thickness). The HU of the water itself at the same point was noted, as well. The mass of each plug was also measured and used to compute its mass density and, from the vendor's elemental composition datasheet, its volumetric electron density. Using these data, stoichiometric calibrations were carried out based on whether the nominal $\rho_{e_{i}}^{\text{rel}}$ values provided by the vendor or those computed by us were used as input.

### C. Slice thickness

A similar HU dataset to that in Materials and Methods section B above was collected in our standard configuration, though using 1.5 mm instead of 3 mm CT slice thickness. Employing the vendor's nominal electron densities in both cases, stoichiometric calibrations were carried out and compared.

### D. Phantom size/plug location

An HU dataset similar to that in Materials and Methods section B was collected in our standard configuration, though placing each plug alternately in the inner hole (6 cm off‐axis) and the outer hole (12 cm off‐axis) of the acrylic holder (Figs. 1(a) and (b)). The tank was then rotated by 90° so that its widest dimension (40 cm) was aligned with the scan plane, and additional data were collected with each plug alternately in the central, inner, and outer holes of the holder. Stoichiometric calibrations were carried out for each of the five variants, employing nominal electron densities in all cases, and compared with that derived from the standard configuration.

### E. Simultaneous data acquisition

The 14 Gammex plugs were distributed throughout the vendor's 33 cm diameter tissue characterization phantom holder, inserted fully into their respective holes (Fig. 1(c)) and scanned simultaneously with 3 mm slice thickness. The HUs of the plugs assessed in the central plane through the phantom were used to perform the stoichiometric calibration, employing nominal electron densities in both cases. This was compared with the calibration undertaken in the standard configuration.

### F. Plug manufacturer

Calibration curves computed using either the HU measurements from the Gammex plugs or from the CIRS plugs were compared in the standard configuration with the curves resulting from using all data from both vendors' plugs, employing nominal electron densities in both cases.

### G. Fit weighting

Technically, the fitting of measured data to the independent variable (e.g., the quantity on the left‐hand side of Eq. (1)) and the extraction of the free parameters (A,B, and C) should be performed by weighting each data point by the inverse square of the measurement error in the linear regression. However, this consideration may be easily overlooked or neglected, though may be important if these errors have appreciable variation. The commonly employed commercial software Microsoft Excel (Microsoft Corporation, Redmond, WA), for example, doesn't have the option to accommodate weighting directly, but the appropriate weights can be incorporated equivalently by formulating the data to be fitted in accordance with

(5) $$\frac{HU_{i}+1000}{\Delta HU_{i}}=A\left(\frac{\rho_{e_{i}}^{rel}{\tilde{Z}}_{i}^{3.62}}{\Delta HU_{i}}\right)+B\left(\frac{\rho_{e_{i}}^{rel}{\tilde{Z}}_{i}^{1.86}}{\Delta HU_{i}}\right)+C\left(\frac{\rho_{e_{i}}^{rel}}{\Delta HU_{i}}\right),$$

where $\Delta HU_{i}$ is the error in the measurement of $HU_{i}$. Plugs of higher HU tend to be more susceptible to noise (Fig. 2(b)) because of the relative paucity of photons measured by the detectors of the CT scanner owing to higher absorption, thus these data points carry lower weights in the fitting procedure. We therefore investigated whether or not neglecting to weight the input data significantly affected the outcome, using the standard configuration.

### H. Inclusion of high‐density plug

The commercial tissue characterization phantoms studied include metallic substitutes of high‐Z (compared to tissue) (and, hence, high HU) as optional add‐ons. However, it is known that the accuracy of the stoichiometric parameterization deteriorates for such materials.^(22)^ Thus, whether or not the inclusion of the additional datum from an Al plug in the fitting procedure significantly affected the outcome was investigated using the standard configuration.

### I. Fit constraining

Equation (1) can be applied to any set of data comprising measured HUs of materials of known composition in order to extract the three parameters A, B, and C, which are treated implicitly as being independent from one another. However, one can constrain the fit by noting that, for water, HU=1000 and $\rho_{e}^{rel}=1$, by definition. Thus, we also have

(6) $$1000=A{\tilde{Z}}_{w}^{3.62}+B{\tilde{Z}}_{w}^{1.86}+C$$

where the subscript *w* denotes water. Eliminating *C* between Eq. (6) and Eq. (1) gives, in the unweighted case

(7) $$HU_{i}+1000\left(1-\rho_{e_{i}}^{rel}\right)=A\left(\rho_{e_{i}}^{rel}\left[{\tilde{Z}}_{i}^{3.62}-{\tilde{Z}}_{w}^{3.62}\right]\right)+B\left(\rho_{e_{i}}^{rel}\left[{\tilde{Z}}_{i}^{1.86}-{\tilde{Z}}_{w}^{1.86}\right]\right)$$

while eliminating *C* between Eq. (6) and Eq. (5) gives, in the weighted case

(8) $$\frac{HU_{i}+1000\left(1-\rho_{e_{i}}^{rel}\right)}{\Delta HU_{i}}=A\left(\frac{\rho_{e_{i}}^{rel}\left[{\tilde{Z}}_{i}^{3.62}-{\tilde{Z}}_{w}^{3.62}\right]}{\Delta HU_{i}}\right)+B\left(\frac{\rho_{e_{i}}^{rel}\left[{\tilde{Z}}_{i}^{1.86}-{\tilde{Z}}_{w}^{1.86}\right]}{\Delta HU_{i}}\right)$$

Hence the fit can be constrained such that there are two, rather than three, free parameters. For all of the aforementioned HU data measured in the standard configuration for both the Gammex and CIRS plugs, stoichiometric calibrations were therefore performed according to both Eq. (1) (three‐parameter fits) and Eq. (7) (two‐parameter fits) in the unweighted cases, and according to both Eq. (5) (three‐parameter fits) and Eq. (8) (two‐parameter fits) in the weighted cases.

Table 2 summarizes the essential components of the 20 tests referred to in Materials and Methods sections B to I above, each of which was analyzed both with a three‐parameter fit and a two‐parameter fit, yielding a total of 40 calibration curves for intercomparison.

**Table 2:** Summary of the 40 scenarios for which the stoichiometric calibration was conducted

*Test*								
*3‐par fit*	*2‐par fit*	*Plugs / Materials*	*Phantom Material*	*Phantom Width (cm)*	*Plug Hole*	*Slice Thickness (mm)*	*Weighted*	$\rho_{e}^{rel}$
1a	1b	G, W	water	30	center	3	x	nominal
2a	2b	G, W	water	30	center	3	x	calculated
3a	3b	G, W	water	30	center	1.5	x	nominal
4a	4b	G, W	water	30	inner	3	x	nominal
5a	5b	G, W	water	30	outer	3	x	nominal
6a	6b	G, W	water	40	center	3	x	nominal
7a	7b	G, W	water	40	inner	3	x	nominal
8a	8b	G, W	water	40	outer	3	x	nominal
9a	9b	G	solid water	33	distributed	3	x	nominal
10a	10b	G, W	water	30	center	3	×	nominal
11a	11b	C, W	water	30	center	3	×	nominal
12a	12b	C, W	water	30	center	3	×	nominal
13a	13b	G, C, W	water	30	center	3	x	nominal
14a	14b	G, C, W	water	30	center	3	×	nominal
15a	15b	G, W, Al	water	30	center	3	x	nominal
16a	16b	G, W, Al	water	30	center	3	×	nominal
17a	17b	C, W, Al	water	30	center	3	x	nominal
18a	18b	C, W, Al	water	30	center	3	×	nominal
19a	19b	G, C, W, Al	water	30	center	3	x	nominal
20a	20b	G, C, W, Al	water	30	center	3	×	nominal

G=Gammex plugs; C=CIRS plugs; Al=aluminum plug, W=water.

## III. RESULTS & DISCUSSION

### A. Extraction of HU data

Figure 2(b) illustrates the variation of the uncertainty in the measured HU of a plug with the size of the ROI employed in the CT image for five of the Gammex plugs employed in the study, as examples. Given the 28.5 mm plug diameter and 0.98 mm pixel size, the largest square ROI which can be contained within a plug's cross section is 20 pixels on a side. As might be expected on statistical grounds, this sized ROI can be seen to result in the lowest uncertainties in the mean measured HUs of these five plugs. Similar conclusions were drawn from studies with the other plugs also. It was therefore decided that the choice of an ROI of 20×20 pixels would be appropriate uniformly for the determination of the mean HU of all plugs in the various scenarios.

**Figure 3:**
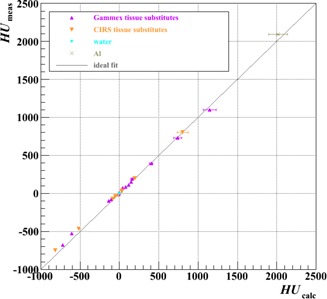
Measured HUs versus those calculated using the results of the fit of Eq. (8) in Test 20b (Table 2). Errors on the calculated HUs were propagated from those on the fit parameters (A and B). The diagonal line indicates what would be ideal agreement.

**Figure 4:**
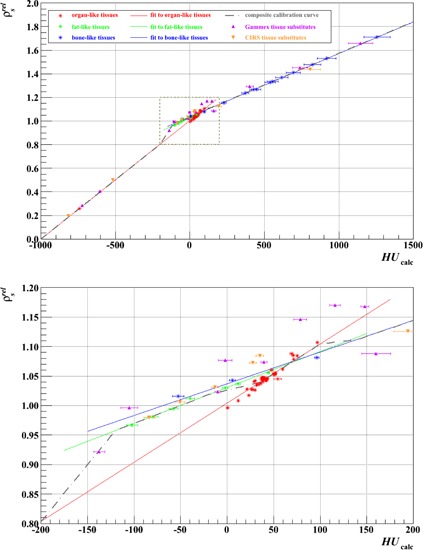
Stoichiometric calibration using the data of Test 20b (Table 2). The composite calibration curve is pieced together from separate fits to the points for organ‐like, fat‐like, and bone‐like tissues. Errors on the calculated HUs were propagated from those on the fit parameters (A and B). The points for the Gammex and CIRS tissue‐surrogate plugs are plotted for comparison with the real tissues. The lower plot magnifies the area of the upper plot in the dashed, boxed region, from ‐200 HU to +200 HU.

Figures 3 and 4 demonstrate, as an example, the procedure adopted to derive the calibration curve for each scenario listed in Table 2, here for Test 20b. Figure 3 displays for all 25 data points the HUs calculated from Eq. (8), based on the fitted *A* and *B* parameters, versus those measured, color‐coded by source; Fig. 4 shows the calculated relative stopping powers (Eq. (4)) versus calculated HUs (Eq. (8)) for both the plugs and the 63 human tissues. For the human tissues, the linear fit through the 42 “organ‐like” points is colored red, through the seven “fat‐like” points is colored green, and through the 14 “bone‐like” points is colored blue. The resulting calibration curve (black, dashed line) was adjudged to track the red curve from ‐1000 to ‐200 HU, the green curve from ‐120 to ‐20 HU, the red curve again from +35 to +100 HU, then the blue curve above 140 HU, with intermediate nodes connected by straight lines.^(13)^ While the composite curve fits the $\rho_{s}^{rel}$ of all human tissue data to within 3% (and is much better in most cases), notably it can deviate from the synthetic tissue substitutes of both vendors by in excess of 5%. Even though manufacturing technologies have evolved over the last two decades, it is evident that a calibration performed directly from tissue substitutes without recourse to the stoichiometric procedure is still, therefore, insufficient (and this was, of course, the reason for its introduction in the first place).

**Figure 5:**
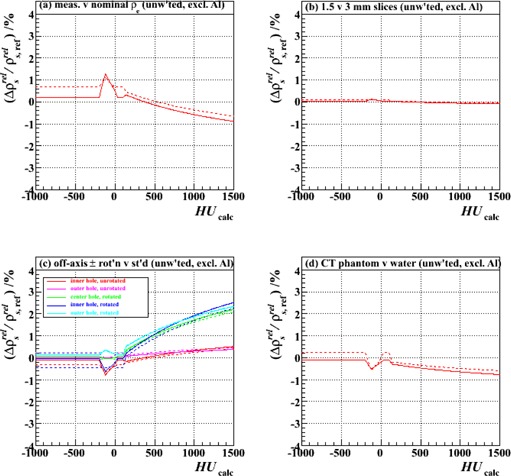
Fractional differences in relative proton stopping power as a function of HU for stoichiometric calibrations carried out with (a) electron densities derived from measurement rather than the vendor's nominal values (Test 2 vs. Test 1), (b) 1.5 mm rather than 3 mm CT slice thickness (Test 3 vs. Test 1), (c) plugs scanned at different off‐axis locations (0, 6 cm and 12 cm) and with the tank in two alternative orientations (presenting 30 cm or 40 cm width), rather than in the center hole and the standard orientation (presenting 30 cm width) (Tests 4‐8 vs. Test 1), and (d) the Gammex tissue characterization phantom with all plugs scanned simultaneously, rather than the water tank with plugs scanned individually (Test 9 vs. Test 1). The results of both two‐ and three‐parameter fits (solid and dashed lines, respectively), all unweighted and excluding the Al plug, are shown.

**Figure 6:**
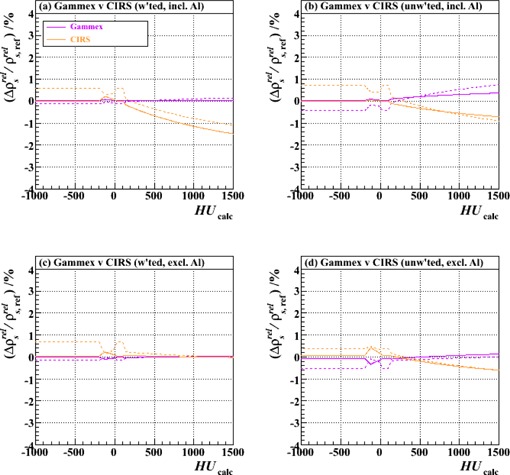
Fractional differences in relative proton stopping power as a function of HU for stoichiometric calibrations carried out with either Gammex or CIRS plugs relative to the combined set of both. The results of using both two‐ and three‐parameter fits (solid and dashed lines, respectively) are shown: (a) weighted fits, including also the Al plug (Tests 16 and 18 vs. Test 20), (b) unweighted fits, including the Al plug (Tests 15 and 17 vs. Test 19), (c) weighted fits, excluding the Al plug (Tests 10 and 12 vs. Test 14), and (d) unweighted fits, excluding the Al plug (Tests 1 and 11 vs. Test 13).

**Figure 7:**
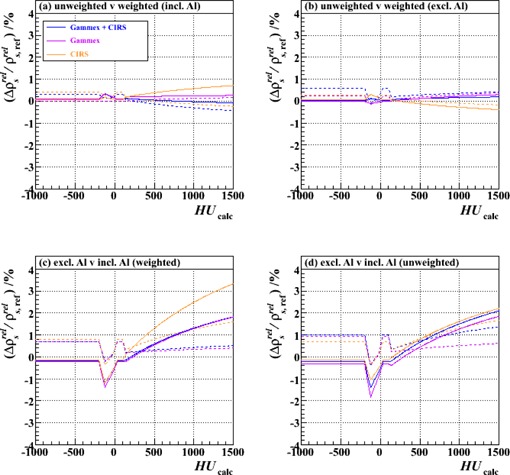
Fractional differences in relative proton stopping power as a function of HU for stoichiometric calibrations carried out with weighted fits compared to those carried out with unweighted fits: (a) including the Al plug (Tests 19, 15 and 17 vs. Tests 20, 16 and 18, respectively), (b) excluding the Al plug (Tests 13, 1 and 11 vs. Tests 14, 10 and 12, respectively); and for calibrations carried out including the Al plug compared to those excluding it: (c) weighted fits (Tests 14, 10 and 12 vs. Tests 20, 16 and 18, respectively), (d) unweighted fits (Tests 13, 1 and 11 vs. Tests 19, 15 and 17, respectively). The results of using either the combined set of Gammex and CIRS plugs or one or other set alone are shown, as are the results of using either two‐ or three‐parameter fits (solid and dashed lines, respectively).

**Figure 8:**
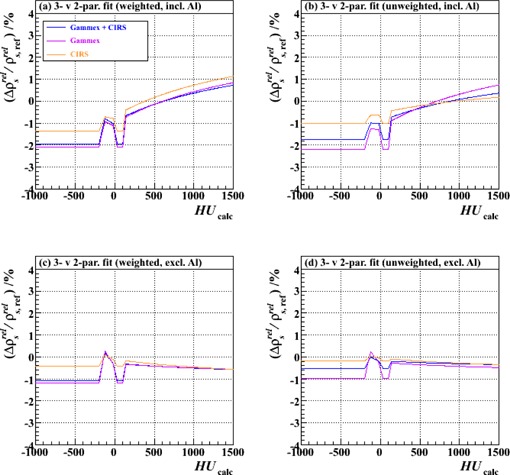
Fractional differences in relative proton stopping power as a function of HU for stoichiometric calibrations carried out with three‐parameter fits compared to those carried out with two‐parameter fits. The results of using either the combined set of Gammex and CIRS plugs or one or other set alone are shown: (a) weighted fits, including also the Al plug (Tests 20, 16 and 18: a vs. b), (b) unweighted fits, including the Al plug (Tests 19, 15 and 17: a vs. b), (c) weighted fits, excluding the Al plug (Tests 14, 10 and 12: a vs. b), and (d) unweighted fits, excluding the Al plug (Tests 13, 1 and 11: a vs. b). “a vs. b” implies a comparison between data analyses performed for a particular test with either a three‐parameter fit (type “a” test, Table 2) or a two‐parameter fit (type “b” test, Table 2).

Figures 5‐8 show relative differences in the calibration curve of $\rho_{s}^{rel}$ as a function of calculated HU based on intercomparisons of pairs of calibration curves, each constructed piecewise by the method described above (and with joins at the same intermediate nodes: ‐200, ‐120, ‐20, +35, +100 and +140 HU) from a specific combination of measurement setup and data processing technique (Table 2) and extending over the typical anatomical range of HUs (‐1000 to +1500). Figure 5 is based on measurements made with just the Gammex plugs, analyzed with unweighted fits; Figs. 6‐8 derive from those made with both vendors' plugs and also the custom‐made Al plug, analyzed with both unweighted and weighted fits.

### B. Electron density

Figure 5(a) demonstrates that, over this span of HUs, the result of calculating the relative electron densities of the plugs from measurements of mass densities and vendor‐provided elemental composition tables for use in Eqs. (1), (4), and (7) yields $\rho_{s}^{rel}$ values (Eq. (4)) that vary by less than ∼1% from those based on the vendor's nominal electron densities. This is true both for two‐paramater (Eq. (7)) and three‐parameter (Eq. (1)) fits. Thus, we conclude that the use of the nominal vendor‐provided $\rho_{s}^{rel}$ values is adequate, assuming the elemental composition tables themselves are reliable (discussed further in Results & Discussion section F below).

### C. Slice thickness

Figure 5(b) illustrates that the calibration procedure is independent of CT slice thickness, and so does not need to be carried out multiple times if this parameter varies across an institution's scanning protocols. All further studies were, hence, conducted with the same 3 mm slice thickness only, in order to minimize noise.

### D. Phantom size/plug location

Figure 5(c) demonstrates that, for a given phantom size, the calibration curve is largely independent of the plug placement within the scanner's field of view (for plugs placed at 0, 6, and 12 cm off‐axis), but that significant deviations may arise, which increase with HU, when the phantom size changes. This is an important point. Although it is not the purpose of our present work to study this variation exhaustively, our measurements in 30 cm and 40 cm width phantoms alone suffice to suggest that, in order to avoid incurring proton range errors in regions of high HU, one may wish to consider implementing multiple (two or more) calibration curves in practice, with the most appropriate one selected in a given situation according to the size of the relevant anatomy. For sites much smaller than 30 cm (e.g., child's extremity) or much larger than 40 cm (e.g., adult's pelvis), the distortions from the baseline curve may be unacceptably large and, if unaccounted for through choice of an appropriate calibration curve selection, the 3.5% range uncertainty margin in treatment planning will likely be inadequate in cases where a large amount of high‐density material is traversed. The finding is the same whether two‐parameter or three‐parameter fits are used.

### E. Simultaneous data acquisition

Figure 5(d) illustrates that when the vendor‐provided 33 cm diameter solid water phantom holder is used and all plugs are scanned simultaneously, as opposed to one by one in the center of a square water‐filled tank of width 30 cm, the variation in $\rho_{s}^{rel}$ across the span of HUs is less than 1%. This is also a useful practical finding, since it implies that a simplified setup up may be used to derive the calibration curve for a particular phantom size. It is also consistent with the results of Fig. 5(c), which showed that plug position within the field of view has little bearing on the outcome, permitting all plugs to be scanned at once at different off‐axis positions. The general applicability of this conclusion, however, likely depends on the adequacy of the user's reconstruction algorithm in removing beam hardening‐related (e.g., “cupping” and “capping”) artifacts; so, although the case here, this may not necessarily always be so.

### F. Plug manufacturer

Figure 6 shows a comparison of calibrations carried out using either the Gammex or CIRS plugs exclusively (together with the corresponding vendors' tables of nominal relative electron density and elemental composition), analyzed with (upper plots) or without (lower plots) the addition of the Al plug, and with (left‐hand plots) or without (right‐hand plots) weighting of the data according to HU measurement uncertainty. In each plot, the result for each independent vendor is shown relative to that based on both vendors' data combined. The mutual compatibility of the different vendor's curves, independent of the method of data handling, serves to provide a degree of comfort in the otherwise tacit assumption that the vendors' nominal tables of electron density and elemental composition for the plugs are reliable. In the high HU region, fits to the CIRS plug data are likely more sensitive to the inclusion of the datum for the Al plug than fits to the Gammex plug data (upper plots) because the former comprise fewer data points, and especially so in this very region. Nevertheless, the deviation between the two never exceeds ~ 1.5% at+1500 HU, so we conclude that the vendors' data are consistent with one another and that an institution can opt to use either with equal confidence (or, indeed, combine both).

### G. Fit weighting

Figures 7(a) and (b) demonstrate that the effects of weighting versus not weighting the HU data according to the measurement uncertainties are small, at least when the independent variable is presented as in Eq. (1) (three‐parameter fit) or Eq. (7) (two‐parameter fit) (i.e., the quantity on the left‐hand side of the equation is linear in ${\text{HU}}_{i}$). However, it should be noted that one could conceivably rearrange these equations any number of ways and still retain mathematical equality (e.g., making $(HU_{i}+1000)/\rho_{e_{i}}^{rel}{\tilde{Z}}_{i}^{3.62}$ the independent variable in Eq. (1), etc.); without including and propagating correctly the weights, the fitting results would be dependent on the precise formulation used. (The weight is proportional to the inverse square of the factor by which ${\mathrm{HU}}_{i}$ is divided, so the example arrangement above would, for instance, be tantamount to weighting the data points implicitly by $(\rho_{e_{i}}^{rel}{{\tilde{Z}}_{i}^{3.62})}^{2}$, skewing the fit strongly in favor of the measurement of plugs with small ${\tilde{Z}}_{i}$ if explicit weights are not properly included.) Since weighting the data requires little extra effort, we therefore advise that it should be incorporated into the procedure to avoid such pitfalls (i.e., Eqs. (5) and (8) should be preferred over Eqs. (1) and (7)).

### H. Inclusion of high‐density plug

Figures 7(c) and (d) illustrate that the inclusion of a high‐density plug can strongly influence the result of the fitting procedure, especially in the high HU region. While the impact of the Al plug on the two vendors' datasets has a similar trend, the size of the effect on the CIRS data may be greater than on the Gammex data because there are fewer other points in the data series and those other points also tend to fall at lower HUs (3 out of 14 Gammex plugs, but only 1 out of 9 CIRS plugs, have HUs >∼200 (see, e.g., Figs. 3 and 4)), as already suggested in Results & Discussion section F. Nevertheless, the likely reason for the observed deviation in both cases is the inadequacy of the stoichiometric parameterization for high‐Z (compared to tissue) materials. The exponents in Eq. (1) (and the others that follow) were obtained by optimization for oxygen for X‐ray energies between 60 and 80 keV;^(21)^ considerable discrepancies have been shown to appear as Z increases.^(22)^ We would, therefore, propose that metallic plugs be avoided when performing the calibration.

### I. Fit constraining

Figure 8 shows that the application of the constraint in Eq. (6) to the fitting procedure affects the outcome of the calibration by at most 2%, and only then for negative or small, positive HUs when the Al plug is included in the fitting procedure. (Note, however, the discussion in Results & Discussion section H.) The trends are similar for both vendors' datasets (and their combination) in each of the data handling approaches. Although these findings may only be of small concern, we would nevertheless recommend the adoption of the constrained (i.e., two‐parameter) fit, since Eq. (6) follows directly from the definition of the Hounsfield unit itself.

## IV. CONCLUSIONS

The calibration of the CT scanner to map Hounsfield units to relative proton stopping powers is a crucial component in assuring the accurate delivery of proton therapy dose distributions to patients. A variety of measurement setups and data handling techniques were investigated in order to assess the robustness of the calibration curve resulting from the stoichiometric method from a user‐specific perspective. From the 40 separate such curves that were derived, the following observations can be made:

(i) tissue surrogates from two alternative vendors (Gammex and CIRS) yield consistent results, giving confidence that the companies' nominal tables of electron density and elemental composition for these materials are, therefore, likely reliable;
(ii) similarly sized anatomies scanned with different slice thicknesses share the same calibration curve;
(iii) the use of multiple calibration curves should be considered if anatomical sites of widely differing size are to be treated;
(iv) in a given scan, the same calibration curve applies equally well throughout all voxels of the image dataset, with minimal error;
(v) the tissue‐surrogate HU data can be taken quickly and efficiently for a given phantom size with a single scan, rather than from many separate scans of individual plugs;
(vi) the use of metallic materials (i.e., high‐Z compared to tissue) should be avoided; and
(vii) fits should be conducted based on the statistical errors on the HU measurements and constrained so as to characterize the X‐ray spectrum of the CT scanner by only two parameters rather than three (i.e., Eq. (8) should be preferred).

It is important to note, however, that observations (iv) and (v) may be made only if the beam hardening artifact reduction algorithm in the user's image reconstruction software is adequate, as was found to be the case here.

**Figure 9:**
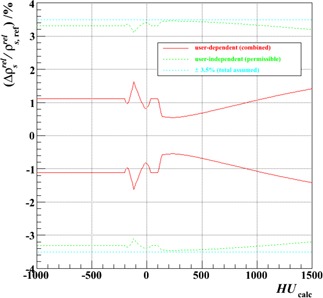
Combined user‐dependent fractional uncertainty in relative proton stopping power as a function of HU for the stoichiometric calibration, together with the implied, user‐independent uncertainty permissible if an overall uncertainty not exceeding 3.5% is to be maintained when the two are summed in quadrature. Both uncertainties are displayed as ± bands, together with the total assumed ±3.5% limit.

Based on these findings, Test 14b (Table 2) may be singled out as our favored scenario. Using this as a baseline comparator and summing in quadrature the fractional differences in relative proton stopping power as a function of HU plotted in Figs. 5(a)‐(d), 6(c), 7(b), and 8(c), yields what may be regarded as a combined user‐dependent uncertainty from this analysis, which is shown in Fig. 9. For this purpose, the Al plug data have been ignored (observation (vi)) and phantom size variations have been omitted, since separate anatomical‐size‐dependent calibrations can, in principle, be constructed and used selectively. Also shown in Fig. 9 is its complement—the residual uncertainty which, when added in quadrature to this user‐dependent uncertainty, results in an overall 3.5% range uncertainty. (Implicitly included in this would be any component from anatomical‐size‐dependence, therefore.) Across this clinically relevant span of HUs it is evident that the user‐dependent variants identified here contribute uncertainties that are well contained within the total 3.5% range uncertainty commonly employed in proton therapy treatment planning margins. As would be hoped, therefore, so long as the caveats identified here are heeded, such margins are not compromised by the precise details of a given institution's acquisition and handling of the calibration data.
